# An organic neurophysiological tool for neuronal metabolic activity monitoring

**DOI:** 10.1063/1.5050170

**Published:** 2018-12-19

**Authors:** A. Spanu, M. T. Tedesco, L. Martines, S. Martinoia, A. Bonfiglio

**Affiliations:** 1 Department of Electrical and Electronic Engineering, University of Cagliari, Via Marengo, 09123 Cagliari (CA), Italy; 2 Center for Materials and Microsystems, FBK-“Bruno Kessler” Foundation, Via Sommarive 18, 38123 Povo (TN), Italy; 3 Department of Bioengineering, Robotics and System Engineering, University of Genoa, Via all'Opera Pia 13, 16145 Genova (GE), Italy

## Abstract

Monitoring cell metabolism *in vitro* is considered a relevant methodology in several scientific fields ranging from fundamental biology research to neuro-toxicology. In the last 20 years, several *in vitro* neuro-pharmacological and neuro-toxicological approaches have been developed, with the intent of addressing the increasing demand for real-time, non-invasive *in vitro* systems capable of continuously and reliably monitoring cellular activity. In this paper, an Organic Charge Modulated Field Effect Transistor-based device is proposed as a promising tool for neuro-pharmacological applications, thanks to its ultra-high pH sensitivity and a simple fabrication technology. The preliminary characterization of this versatile organic device with primary neuronal cultures shows how these remarkable properties can be exploited for the realization of ultra-sensitive metabolic probes, which are both reference-less and low cost. These features, together with the already assessed capability of this sensor to also monitor the electrical activity of electrogenic cells, could provide important advances in the fabrication of multi-sensing lab-on-chip devices, thus opening up interesting perspectives in the neuro-pharmacological field.

## INTRODUCTION

In the field of *in vitro* whole cell biosensors, the cellular metabolic activity is undoubtedly one of the most important parameters to monitor. In fact, cellular metabolism is very sensitive to various stimuli, such as drug administration and electrical stimulation, making it a particularly accurate way for assessing the culture state in a whole set of possible applications.^1,2^ Different parameters can give a measure of the cellular metabolic activity, namely, glucose consumption and lactate production,^3,4^ oxygen uptake due to cellular respiration,^5^ and extracellular pH variations. Among these parameters, monitoring the medium acidification caused by the extracellular accumulation of acidic byproducts is a convenient and relatively easy approach. In particular, when considering the metabolic-induced extracellular acidification, it is possible to discriminate between aerobic and anaerobic conditions. Under the former condition, glucose is converted (via pyruvate and acetyl coenzyme A, in a process involving glycolysis followed by the citric acid cycle and oxidative phosphorylation) into CO_2_ producing energy (this pathway is also called respiration), while under the latter condition, glucose is converted into lactate and energy (via pyruvate, which is converted into lactate by lactate dehydrogenase).^6,7^ In the recent past, several methods have been explored in order to meet the important requirement of reliably monitoring cell metabolism, including optical methods,^8^ functionalized electrodes,^9–12^ ISFET (Ion Sensitive Field Effect Transistor)-based devices,^13–15^ and systems based on the LAPS (Light-Addressable Potentiometric Sensor), such as the Cytosensor^®^ Microphysiometer.^16,17^ In fact, cells are highly sensitive to pH in the surrounding medium, and pH variations can induce important modification of the physiological state of the culture, thus constituting a very important parameter to consider during whatsoever electrophysiological and/or pharmacological experiment.

Despite the numerous and very interesting integrated approaches,^18–20^ the goal of having a convenient and possibly disposable multi-sensing platform for such an application is yet to be achieved. This is mainly due to the cost of the devices and the rather complicated approaches employed so far. These aspects are quite important in view of the increasing demand of low-cost, highly efficient, and disposable cell-to-chip interface systems for high-throughput *in-vitro* toxicity assays and pharmacology.

In the specific case of neuronal assemblies, besides the cell metabolism, the electrical activity is also of great interest, particularly in all those experimental approaches in which fundamental mechanisms of drug addition and/or the response of neurons to toxic substances are addressed.^21–24^ In fact, the simultaneous monitoring of the metabolic and the electrical activity during electrophysiological tests increases the amount of information that can be obtained, thus giving scientists the possibility to correlate different aspects of such a complex system.

With the aim of developing a highly efficient and possibly disposable tool for high-throughput *in vitro* toxicity assays and pharmacology, a monitoring system based on an organic transistor has been developed, with the said system including an ultra-sensitive pH sensor specifically tailored for cellular applications. This paper is focused on the preliminary characterization of this device in neurons' metabolism monitoring.

## EXPERIMENTAL RESULTS AND DISCUSSION

**FIG. 1.**
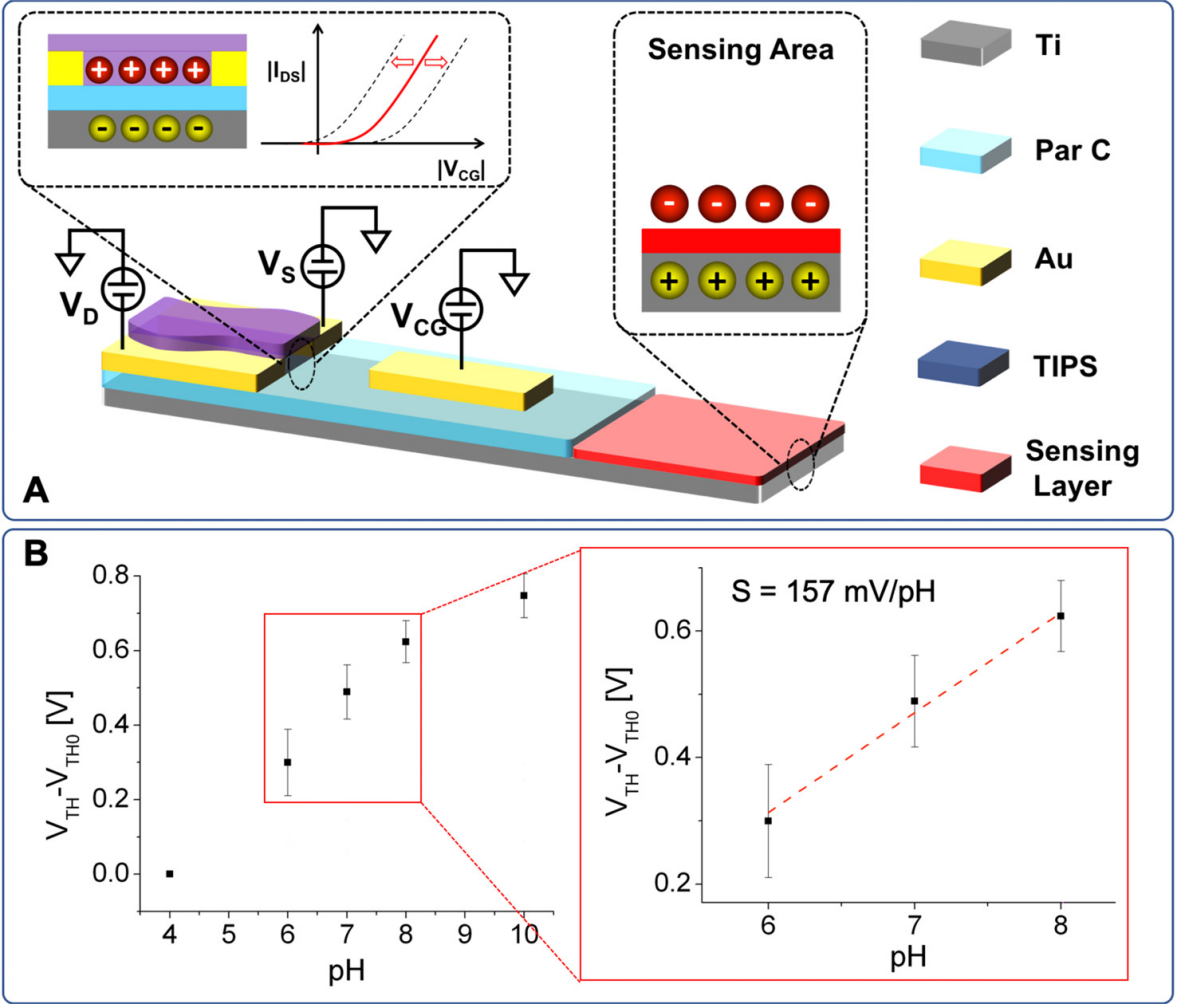
(a) Cross-section of an OCMFET device. The transduction principle is related to a threshold voltage variation induced by the presence of a charge on the sensing area. (b) Characterization of a pH-sensitive OCMFET (sensing area: 4.5 × 10^–2^ cm^2^) in the range of 4–10. The device sensitivity (S = 157 mV/pH) has been extrapolated from the linear part of the calibration curve (i.e., in the range of 6–8).

The organic sensor that has been used and optimized in this work is an Organic Charge Modulated FET (OCMFET). The OCMFET is a peculiar organic thin film transistor which, thanks to the double-gated structure and its simple transduction mechanism, has lately gained a considerable amount of interest in the sensing and biosensing field. Using this device, it was in fact possible to obtain high-sensitive and reference-less sensors such as, just to mention the latest works, sensors for monitoring electrogenic cells' electrical activity,^25^ multimodal tactile sensors,^26^ and DNA-based biosensors.^27^ Its transduction mechanism relies on the modulation of the threshold voltage of the transistor caused by the presence of a charge, Q_SENSE_, in a specific part of the device called the sensing area

$$\Delta V_{TH}\propto -\frac{Q_{\text{SENSE}}}{C_{TOT}},$$

where C_TOT_ is the sum of all the capacitances in the structure. The cross-section of an OCMFET device, together with the materials and the transduction principle, is depicted in Fig. 1(a).

In the specific case of metabolic activity monitoring, a recently developed ultrasensitive OCMFET-based pH sensor has been employed.^28^ It is worth mentioning that this device shows sensitivities beyond the Nernst limit thanks to the amplification effect due to its double-gated structure, with this feature offering a further advantage if compared to classic FET-based pH sensors, because convenient design rules can be defined in order to obtain a device with the desired sensitivity. Another interesting property of this organic transistor-based sensor is that its pH sensitivity is obtained using a simple, yet effective, physical modification of the sensing area as thoroughly described in the Methods section. In Fig. 1(b), the characterization of a pH-sensitive OCMFET is shown. The device sensitivity (S = 157 mV/pH in this specific case) has been extrapolated from the linear region (i.e., between pH 6 and 8), as indicated in the inset.

**FIG. 2.**
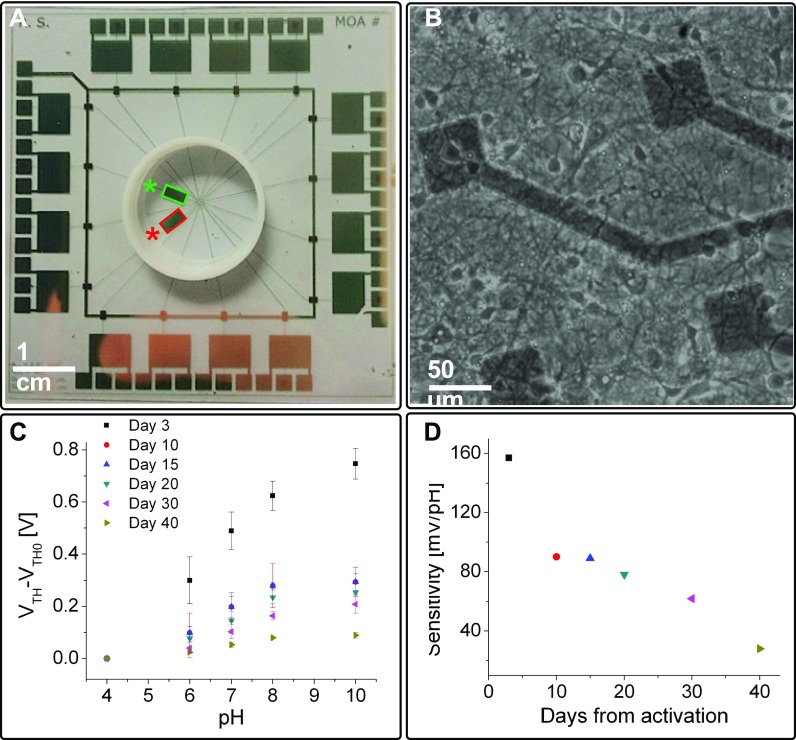
(a) A micro-OCMFET array with a pH-sensitive channel (red) and a pH-insensitive channel (green). The sensing area is 4.5 × 10^–2^ cm^2^ for all the devices. (b) Healthy hippocampal neurons cultured onto the device sensing areas after 15 days *in vitro*. (c) pH characterization of a pH-sensitive OCMFET over a period of 40 days and (d) relative sensitivity.

To meet the requirements of the desired application (i.e., the monitoring of neuronal metabolic activity), it was essential to test the stability of the pH sensitivity of the device over time. Figures 2(c) and 2(d) show the pH response of the test device shown in Fig. 1(b) during 40 days, together with its sensitivity during the same period. The experiment has been conducted by spotting 100 *μ*l of several buffer solutions at different pH values (namely, 4, 6, 7, 8 and 10) onto the sensing area and extrapolating the threshold voltage shift induced by the protonation of the superficial groups of plasma activated Par C. This characterization has been repeated after 3, 10, 15, 20, 30, and 40 days after the activation. The device showed, as expected, a gradual decrease in the sensitivity, due to the nature of the sensing layer. Thisdecrease in the sensitivity can be entirely ascribed to the sensing layer degradation, given the excellent stability of the employed transistor to incubation conditions, as shown in Fig. S2 and as previously demonstrated in Ref. 29. However, despite the afore-mentioned degradation, the sensitivity maintains a relatively high value during at least 3–4 weeks, which is the typical time window of neuro-electrophysiological *in vitro* experiments.

Furthermore, in order to demonstrate the feasibility of the proposed approach for cell monitoring, a particular version of the OCMFET specifically designed for *in vitro* cellular applications, called the Micro-OCMFET Array (MOA), has been employed. The system includes, in the current configuration, 16 OCMFETs, out of which 14 have been designed for monitoring the cells' electrical activity.^25^ As shown in Fig. 2(a), one of the remaining two transistors has a pH-sensitive sensing area, while the other one is pH-insensitive, with the latter sensor being a reference for pH monitoring. The devices have been preliminarily tested using hippocampal neurons from rat embryos, and the cultures have been maintained 15 Days *in vitro* (i.e., 15 DIV) in order to obtain interconnected neuronal networks [cf. Fig. 2(b)] with spontaneous electrophysiological activity. To confirm the electrical activity of such neuronal cultures, hippocampal neurons from the same preparation have been cultured onto a standard Multichannel Systems MEA and their activity has been recorded, as shown in Fig. S3 (supplementary material).

With the intent of mimicking a possible toxicological experiment, the output currents of the two OCMFETs (the “active” and the “inactive” one) present on each MOA were monitored simultaneously during two different phases, clearly distinguishable one from the other, namely, the basal activity and the activity after the administration of 25 *μ*M of bicuculline (BIC). This compound is in fact known for its “boosting” effect on the neuronal electrical activity due to its well-known inhibitory effect on GABA A receptors,^30^ which also affects the metabolic rate of the whole culture, accelerating it, due to the increased neuronal glucose consumption.^31^ After this first characterization of the system, the medium is washed out and replaced with a medium containing a very high dose (10 *μ*M) of tetrodotoxin (TTX), which, thanks to its inhibiting effect on the fast voltage gated sodium channels, determines a cessation of the cells' electrical activity and an abrupt slowing down of their metabolism, which eventually leads to cellular death.^32–35^ After 10 minutes, the previously described experiment (basal + BIC) is then repeated. The same protocol has been replicated with four MOAs, two with the cells (MOA1 and MOA2) and two without the cells (Blank1 and Blank2), in order to evaluate a possible nonspecific response of the sensors. For all the experiments, right before the beginning of the experimental session, the regular medium was replaced with a small volume (100 *μ*l) of a low buffered one (i.e., a low buffered Krebs-Ringer solution, whose formulation is shown in the Methods section) in order to be able to monitor the small pH variations induced by the cell activity.

**FIG. 3.**
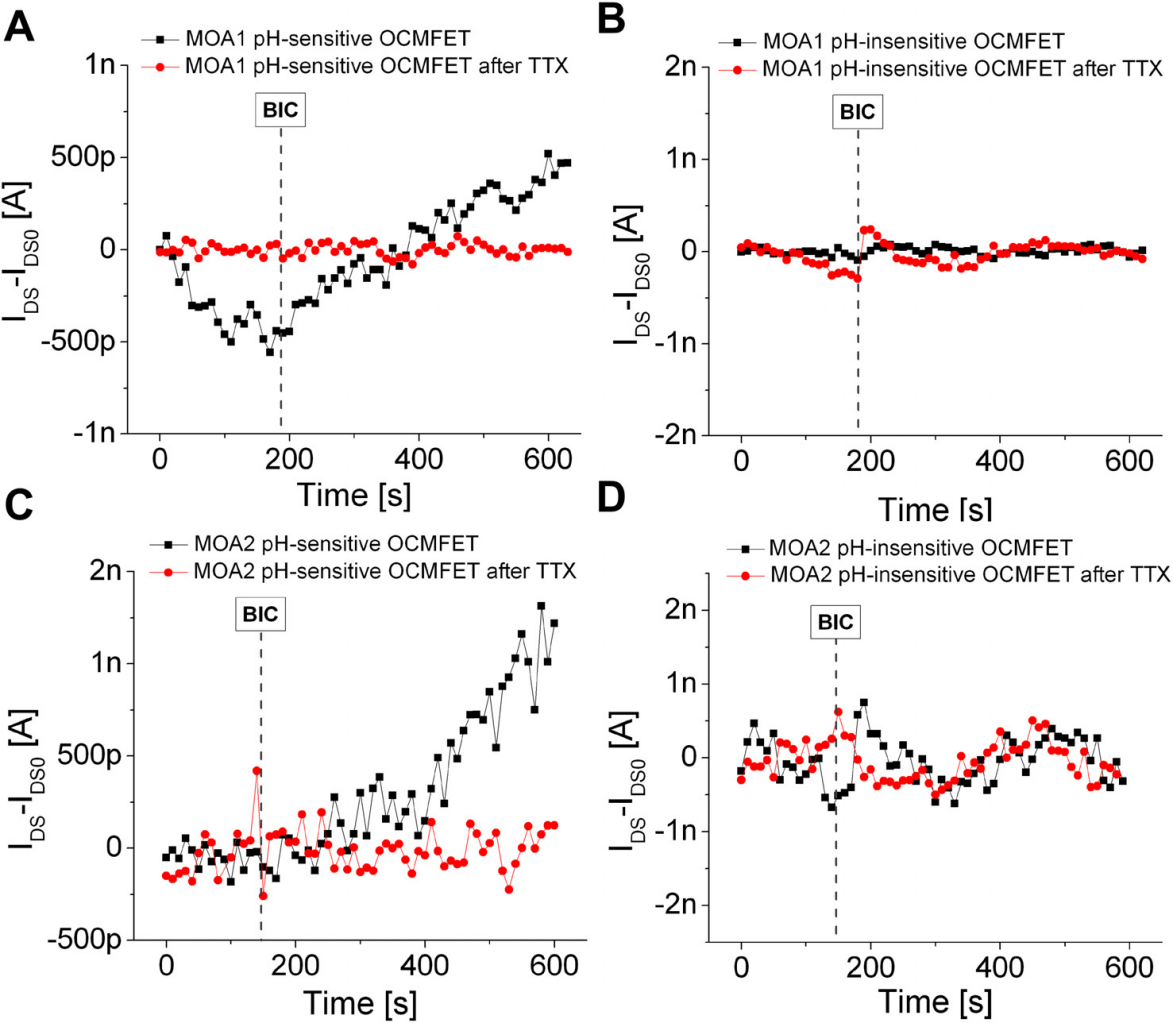
Experimental results. Response of the pH-sensitive and pH-insensitive channels of MOA1 [panels (a) and (b), respectively] and MOA2 [panels (c) and (d)] to the BIC (25 *μ*M) before and after the addition of a high dose (10 *μ*M) of TTX. After the TTX addition, the pH-sensitive channels start behaving like the pH-insensitive ones (no difference between the activity before and after the BIC addition) due to the TTX-induced cellular death. For all the currents, the bias stress has been subtracted in order to enhance the readability.

In Figs. 3(a) and 3(b), the normalized current of the pH-sensitive and pH-insensitive channel of MOA1 is shown, while Figs. 3(c) and 3(d) show the same experiment performed with MOA2. The pH-sensitive channels of both MOA1 and MOA2 showed a clear response to the addition of the BIC, thus indicating a sudden change in the extracellular medium acidification rate (AR). In particular, upon the addition of the BIC (which is administered after a few minutes of basal activity), the current slope rapidly changes, thus indicating a shift of the transistor current toward more positive values, which means, by considering the sensor' transduction mechanism reported in Ref. 28, a switching-off of the transistor itself. This behavior can be ascribed to the acidification of the culture medium, while, in contrast, a shift of the current toward more negative values (i.e., a switching-on of the transistor) can be interpreted as a basification of the extracellular medium. The initial basification that can be noticed during the basal phase can be explained with the low-buffered medium basification due to the momentary re-assessment of cellular steady state conditions (which undergo a perturbation during the medium change and the device transfer from the incubator to the experimental setup). Interestingly enough, it can be noticed that after the addition of a very high dose of TTX, the pH-sensitive OCMFET response is completely inhibited, whilst the pH-insensitive devices remained unaffected by the addition of the BIC in either cases, and this effect can be explained by the metabolic activity inhibition induced by the TTX.

**FIG. 4.**
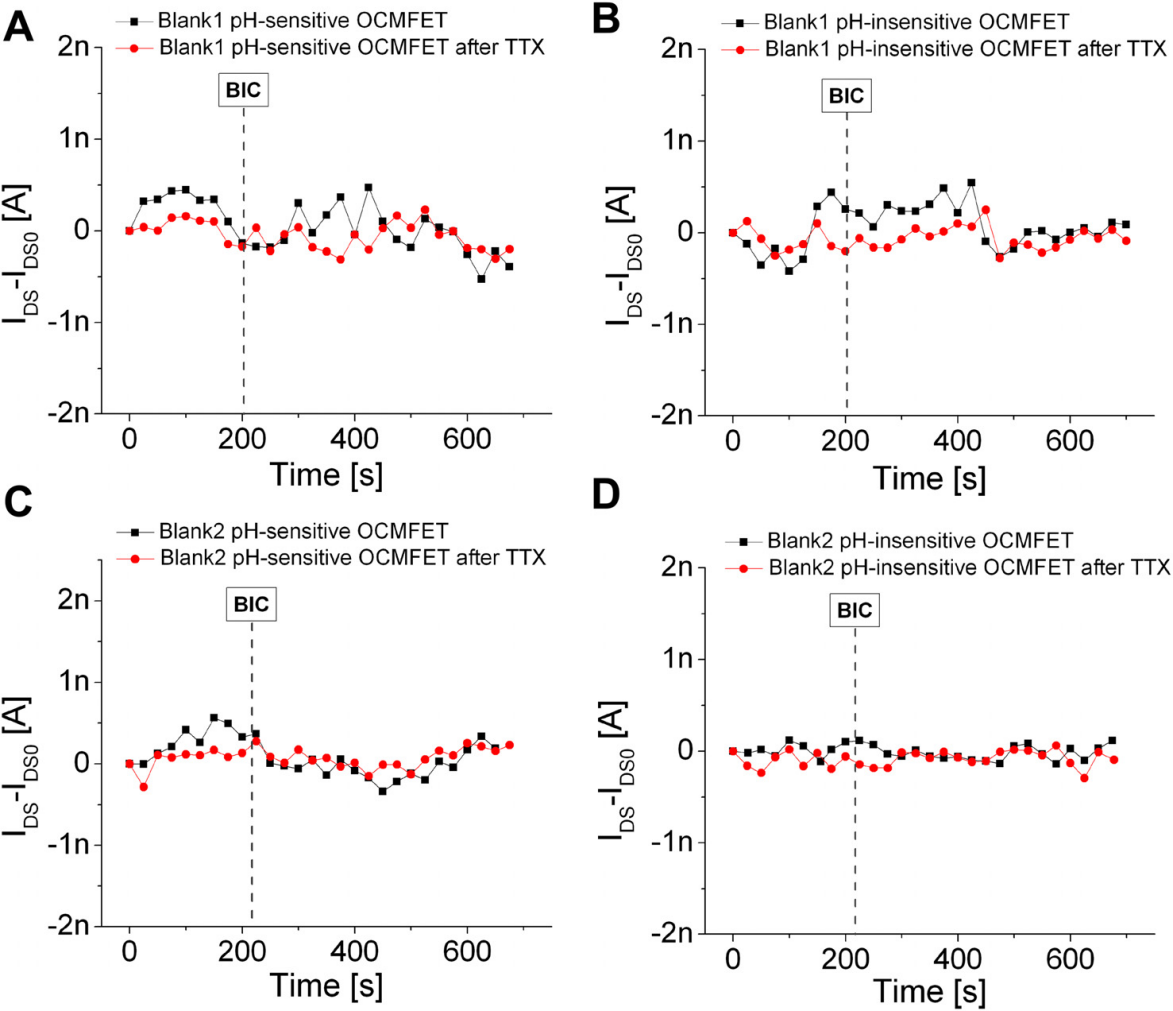
Experiments with the blanks. Response of the pH-sensitive and pH-insensitive channels of Blank1 [panels (a) and (b), respectively] and Blank2 [panels (c) and (d)] to the BIC (25 *μ*M). The same experimental protocol has been applied to the two blanks in order to evaluate the spontaneous, nonspecific oscillations of the transistors' output current. No difference can be appreciated between the activity before and after the addition of the BIC in both TTX and TTX-free experiments. For all the currents, a bias stress subtraction has been performed.

The very same experiment has been repeated with the two identical blanks (i.e., devices without the cells cultured onto the sensing area), obtaining no significant current variations in the four phases (namely, basal, BIC, basal after TTX, and BIC after TTX), as shown in Figs. 4(a)–4(d). In the supplementary material (Fig. S4) is also shown how the BIC effect cannot be appreciated when the protocol is applied to the very same devices and cell cultures using a standard buffered medium (namely, Neurobasal™ from Termo Fisher).

Starting from the equation of a p-type transistor in saturation and using the sensors' parameters (which are shown in Table I), it is possible to estimate the acidification rate (AR) for the two devices, according to the following formula:

$$\Delta I_{DS\_BIC}=K{\left(-V_{GS}+V_{TH\_BIC}\right)}^{2}-K{\left(-V_{GS}+V_{TH0}\right)}^{2}\Rightarrow V_{TH\_BIC}=\frac{\sqrt{\frac{{\Delta I_{DS\_BIC}+K\left(-V_{GS}+V_{TH0}\right)}^{2}}{K}}}{{-V}_{GS}},$$ (1)

with being V_TH_BIC_ the threshold voltage after 5 min of BIC-mediated activity. From Eq. (1), it is possible to derive the relative threshold voltage variation

$${\Delta V}_{TH}={V_{TH\_BIC}-V}_{TH0}.$$ (2)

**TABLE I.** Parameters of the two MOAs employed for the experiments (mean and standard deviation over 3 extrapolations).

	μ (cm^2^/V s)	V_TH0_ (V)	I_ON_/I_OFF_	Δ I_DS_BIC_ (nA)
MOA1	(9 ± 0.1) 10^−2^	2.6 ± 0.1	125 ± 10	−0.9
MOA2	(2 ± 0.04) 10^−1^	1.3 ± 0.15	250 ± 25	−1.25

From the threshold voltage variation, it is possible to estimate the acidification rate by simply taking into account the device sensitivity S = 90 mV/pH [which has been extrapolated from the characterization of an identical test device as previously shown in Fig. 2(d)]

$$\text{AR}=\frac{{\Delta V}_{\text{TH}}}{\text{S*min}}.$$

The obtained estimations of the acidification rates are 0.0027 and 0.0013 pH/min for the pH sensitive channels of MOA1 and the MOA2, respectively (the electrical characterization of the two transistors is shown in Fig. S1). These values are in line with typical acidification rate values for *in vitro* metabolic experiments.^14^

## CONCLUSIONS

During the last five years, the OCMFET approach gave rise to a whole set of novel solutions in applications that did not belong, until the introduction of this double gated plastic device, to the organic electronics domain, including electrophysiology and pH sensing above the Nernst limit. In this work, the potentials in (neuro-)pharmacology and toxicology have been explored and preliminary confirmed; this extends the number of possible fields of application of the OCMFET, thus opening up the unprecedented possibility to obtain low-cost, reference-less, and multisensing ultra-sensitive whole-cell based biosensors. The proposed device is in fact able to monitor in real time the small pH variations induced by cell metabolic activity *in vitro*, thanks to its peculiar structure and a very simple physical modification of the sensing area. These features, together with the capability of monitoring also the electrical activity of electroactive cells using the very same structure and materials, make the OCMFET an ideal candidate for the realization of novel lab-on-a-chip platforms for *in vitro* pharmacology and network electrophysiology. Further studies are in progress in the simultaneous recording of electrical and metabolic activities of cellular cultures.

## METHODS

### Device fabrication

The devices tested in this work have been fabricated onto a 250 *μ*m-thick polyethylene terephthalate (PET) substrate. A 100 nm-thick Titanium layer has been employed as the floating gate material, while the native metal oxide and a thin (150 nm) Parylene C film have been chosen for the device gate dielectric. The drain, source, and control gate contacts are then patterned starting from an evaporated gold layer (100 nm). After the fabrication of the bottom-gate/bottom contact device structure, a droplet (1 *μ*l) of a solution of 6,13-Bis(triisopropylsilylethynyl)pentacene (TIPS Pentacene) in anisole (1 wt. %) is drop-cast directly over the channel area. The whole structure is eventually encapsulated with a layer (approximately 500 nm) of Parylene C, thus obtaining a 650 nm-thick layer onto the sensing area (150 nm from the first deposition and 500 from the encapsulation). For all the reported devices, the sensing area was 4.5 × 10^−2^ cm^2^. A careful cleaning of the chip surface with ethanol, acetone, and deionized water precedes every step of the process.

The sensing layer of the pH sensitive channel is obtained by selectively activating the Parylene C layer over the sensing area of only one of the two pH OCMFET devices by exposing it to oxygen plasma (using a patterned layer of a positive resist which is eventually removed after the plasma activation). This process allows obtaining a simultaneous activation of the layer itself according to Ref. 26. The initial 650 nm-thick Parylene C layer, due to the plasma treatment (power: 200 W; operative pressure: 0.5 mbar; exposure time: 6 min), is thinned down to approximately 300 nm. The same treatment has been performed onto all the reported devices. As the last fabrication step, a 3D printed ring (2 cm wide and 7 mm tall) is glued onto the substrate using polydimethylsiloxane (PDMS) in order to delimit the cell culture area.

### Experimental setup

The experimental setup consists of a custom multichannel (16 channels) readout and conditioning electronics, in which two channels out of 16 are specifically dedicated to pH monitoring, each “pH channel” consisting of two main blocks, namely, an I/V converter with a 1 MΩ feedback resistor and a 3rd order low pass Butterworth filter with a cut-off frequency of 10 Hz. The circuit is connected to a Multichannel Systems acquisition board for A/D conversion, acquisition, and storage (www.multichannelsystems.com). The cultures are maintained in a controlled environment (37 °C and a continuous CO_2_/air flux) during the whole experiment. All the measurement sessions were carried out inside a Faraday cage in order to minimize the electrical environmental noise on the system. In all the measurements, the bias stress has been subtracted in order to better show the slight current variations caused by the acidification/basification of the extracellular medium.

### Cell cultures

In order to isolate and culture the fetal hippocampal neurons, rat embryos at day 18 were anesthetized by exposure to CO_2_ and sacrificed. Heads were collected in Ca^2+^/Mg^2+^-free Hank's balance salt solution with 10 mM HEPES [4-(2-hydroxyethyl)-1-piperazineethanesulfonic acid], the same solution in which the whole dissection took place. The brains were then removed from the skull, and the hippocampuses were isolated from the single hemispheres, which were positioned with the ventral aspect facing up. The tissue was then removed and transferred in a sterile conical tube where it was dissociated in 0.125% of Trypsin/Hank's solution containing 0.05% of DNAse (D-5025 Sigma-Aldrich) for 15–18 min at 37 °C. The supernatant solution was removed, and the enzymatic digestion was stopped by adding 10% fetal bovine serum (FBS) in Neurobasal medium for 5 min. Medium with FBS was removed and replaced with culture medium Neurobasal supplemented with B27, 1% glutamax, and gentamicin 10 *μ*g/ml (Gibco Invitrogen). Cells were plated onto the MOA culture area at a total density of 1 × 10^5^ cells in each device. The cultures, containing both glia and neurons, were incubated at 37 °C in a humidified 5% CO_2_ incubator, and after initial plating, half of the medium was exchanged with fresh medium every 3–4 days.

Right before the experimental session, and in order to be able to detect the low pH variations induced by the network activity, the extracellular medium (800 *μ*l) has been replaced with 100 *μ*l of a low-buffered Krebs-Ringer solution with the following formulation: 135 mM NaCl, 2.8 mM KCl, 1 mM MgSO_4_, 1.3 mM CaCl_2_, 10 mM glucose, and 1 mM HEPES. The solution is adjusted to pH 7.4.

### Ethics approval

Ethics approval is not required.

## SUPPLEMENTARY MATERIAL

See supplementary material for the electrical characterization of the pH-sensitive OCMFET devices and for more information supporting the employed experimental approach.
